# CHARACTERIZING AVOIDABILITY OF NURSING HOME RESIDENTS: COMPARING THE CLAIMS-BASED ALGORITHM AND NURSE ASSESSMENT

**DOI:** 10.1093/geroni/igac059.1184

**Published:** 2022-12-20

**Authors:** Justin Blackburn, Jennifer Carnahan, Susan Hickman, Greg Sachs, Kathleen Unroe

**Affiliations:** IU Richard M. Fairbanks School of Public Health at IUPUI, Indianapolis, Indiana, United States; Indiana University Center for Aging Research, Regenstrief Institute, Inc., Indianapolis, Indiana, United States; Indiana University School of Nursing, Indianapolis, Indiana, United States; Indiana University (IU) Center for Aging Research, Regenstrief Institute, Inc., Indianapolis, Indiana, United States; Indiana University Center for Aging Research, Regenstrief Institute, Inc., Indianapolis, Indiana, United States

## Abstract

The elevated risks associated with transferring nursing home residents to the hospital are problematic, but identifying which transfers can be avoided is complex. Using billing claims to determine “avoidability” based on hospital discharge diagnostic codes ignores resource constraints, clinical comorbidities, and asymmetrical information between nursing home staff making the transfer decision at the onset of clinical changes and hospital billing departments following treatment and diagnostic procedures. Conversely, relying on clinical staff assessments at the time of transfer may be an impractical and resource-intensive strategy to drive payment reform and improve quality. Using Medicare claims data representing emergency department and hospitalization transfers from 38 nursing facilities in Indiana from 2016-2020, we compared classification of transfers using a claims-based algorithm and trained nurse assessments of avoidability. Among 960 transfers, nurses judged 48.4% were potentially avoidable while 30.8% were classified as such using claims data. Of concordant assessments, 15.3% were avoidable and 36.0% as not avoidable. Of discordant assessments, 33.1% were judged avoidable by nurses only and 15.5% via the claims-based algorithm (Kappa=0.0153). Discordance was most frequent among transfers with heart failure (64%, n=42), psychosis (74.5%, n=34), acute renal disease (50%, n=28); and lowest among urinary tract infections (31.3%, n=64). No resident demographic or clinical characteristics were associated with discordance (age, race, sex, cognitive function scale, activities of daily living, or CHESS scale). High discordance in determining avoidability may be driven by presentation of symptoms or other condition-specific factors. Policies to reduce avoidable hospitalizations must not rely on overly simplistic approaches for identification.

